# Polymorphic Protective Dps–DNA Co-Crystals by Cryo Electron Tomography and Small Angle X-Ray Scattering

**DOI:** 10.3390/biom10010039

**Published:** 2019-12-26

**Authors:** Roman Kamyshinsky, Yury Chesnokov, Liubov Dadinova, Andrey Mozhaev, Ivan Orlov, Maxim Petoukhov, Anton Orekhov, Eleonora Shtykova, Alexander Vasiliev

**Affiliations:** 1 National Research Center “Kurchatov Institute”, Akademika Kurchatova pl., 1, 123182 Moscow, Russia; chessyura@yandex.ru (Y.C.); orekhov.anton@gmail.com (A.O.); a.vasiliev56@gmail.com (A.V.); 2 Shubnikov Institute of Crystallography of Federal Scientific Research Centre “Crystallography and Photonics” of Russian Academy of Sciences, Leninskiy prospect, 59, 119333 Moscow, Russia; lubovmsu@mail.ru (L.D.); a.a.mozhaev@gmail.com (A.M.); desueagle@gmail.com (I.O.); maxim@embl-hamburg.de (M.P.); eleonora.shtykova@gmail.com (E.S.); 3 Moscow Institute of Physics and Technology, Institutsky lane 9, 141700 Dolgoprudny, Moscow Region, Russia; 4 Shemyakin-Ovchinnikov Institute of bioorganic chemistry of Russian Academy of Sciences, Miklukho-Maklaya, 16/10, 117997 Moscow, Russia; 5 Frumkin Institute of Physical Chemistry and Electrochemistry of Russian Academy of Sciences, Leninsky prospect, 31, 119071 Moscow, Russia

**Keywords:** biocrystallization, Dps, DNA, co-crystals, cryo-electron tomography, small-angle X-ray scattering

## Abstract

Rapid increase of intracellular synthesis of specific histone-like Dps protein that binds DNA to protect the genome against deleterious factors leads to in cellulo crystallization—one of the most curious processes in the area of life science at the moment. However, the actual structure of the Dps–DNA co-crystals remained uncertain in the details for more than two decades. Cryo-electron tomography and small-angle X-ray scattering revealed polymorphous modifications of the co-crystals depending on the buffer parameters. Two different types of the Dps–DNA co-crystals are formed in vitro: triclinic and cubic. Three-dimensional reconstruction revealed DNA and Dps molecules in cubic co-crystals, and the unit cell parameters of cubic lattice were determined consistently by both methods.

## 1. Introduction

During the last two decades, the formation of protective Dps–DNA complexes in stress-induced cells has attracted the attention of many scientific groups, among which the most famous and remarkable are works of A. Minsky and coauthors, who were among the first to publish experimental evidence of this phenomenon (e.g., [1,2,3]. The response to stress of living cells is expressed in their transition to the stationary phase and rapid increase of intracellular synthesis of specific histone-like Dps protein (DNA-binding protein from starved cells), which binds DNA to protect the genome against such deleterious factors as thermal stress, irradiation, toxicity, chemical shock, and oxidative stress. As a result, highly ordered co-crystals are formed with the sole purpose of protecting the DNA from damage [4,5].

Dps is a multifunctional histone-like protein which combines the ferroxidase activity and the ability to bind DNA nonspecifically. Generally, the bacterial histone-like proteins are associated with a nucleoid, maintaining structural integrity and being involving in such DNA-dependent processes as transcription, recombination, replication, or any other. Importantly, histone-like proteins have multiple functions in stress resistance [6]. Dps is the only DNA-binding protein that is produced only in the stationary phase E. coli [4], and it demonstrates the sequence nonspecific DNA binding activity [7,8]. It is well-known that histones being basic protein components of chromatin act as spools around which DNA winds. In our previous work, we proposed a Dps–DNA binding model based on the SAXS data where DNA is bent around Dps confirming histone-like nature of the protein [9]. Dps proteins have been found in the majority of bacterial groups, including archaea [10]. The Dps proteins structure [11] and interaction with DNA [12] have been extensively studied. There is a close evolutionary link between ferritin proteins sequestering iron and Dps proteins [13]. In addition, there are also ferritin-like proteins that possess the functional properties of Dps [14]. The crystalline structure of Dps proteins is presented in [15,16] and it was demonstrated that, similar to ferritins, Dps proteins assemble into oligomers and their three-dimensional shape is absolutely critical to their function [17]. Currently, it was found the diverse functions of Dps genes [5,18,19,20] and different Dps proteins in the same genome. Due to the increase in the number of fully sequenced microbial genomes, many Dps homologs were found, and their structures and functions were clarified [21,22]. It is common amongst bacteria to have more than one Dps gene per genome [23]. A number of studies have demonstrated the Dps expression control at the transcription level [23,24]. It has also been found that expression of these genes may be due to a multitude of sigma factor complexes promoting the transcription of Dps [24,25], for example, msdps1 [25] and msdps2 [23]. That indicates that the evolutionary history of Dps in many genera contains duplicates, losses, and possible lateral acquisitions.

However, the Dps protein is best known as the main structural DNA-condensing factor, which interacts with the bacterial nucleoid via twelve unstructured N-terminal tails containing three lysine and one arginine residues [26]. Formation of Dps–DNA liquid crystal mesophases depends strongly on the protein structure, which in turn depends on environmental conditions, so the dodecameric form is capable of DNA binding and forming of large crystalline arrays, whereas the dimeric or trimeric forms have a decreased protection efficiency [27,28]. In addition, it is important to emphasize the role of the N-terminal domain structure in the process of Dps–DNA co-crystallization. It was shown, for example, that disordered lysine-rich N-terminal region of E. coli Dps extends into the channels at the interfaces between three adjacent Dps dodecamers to mediate DNA binding [15].

The role of the lysine-rich and flexible Dps N-terminus in DNA protection was also demonstrated by Ceci et al. [26]. Moreover, the authors showed the influence of pH in the solution: at pH values beyond the physiological range, the formation of large Dps–DNA complexes is impossible due to deprotonation of lysine residues. Generally, the disordered N-terminal regions of Dps proteins seem to be crucial for the formation of crystalline protein-DNA complexes. It was shown that proteins of the Dps family without positively charged N-termini do not demonstrate binding to DNA [29,30,31]. Buffer composition and pH affect both the structure of the Dps protein itself and the Dps–DNA interaction [27]. It was also demonstrated that Dps is unable to bind DNA directly and Dps–DNA complex formation relies on the ion bridges formed by Mg^2+^ [32,33]. However, our recent results demonstrate a formation of highly ordered Dps–DNA complexes in solution in the absence of the metal ions [9].

Thereby, while the structure of Dps and the conditions of its complexation with DNA were mostly studied [12,15], an actual structure of the Dps–DNA co-crystals under different environmental factors remains uncertain.

Recently, we have collected and analyzed the data of two complementary structural methods, cryo-electron tomography (CET) and synchrotron small-angle scattering (SAXS), and have demonstrated a formation of the central symmetric triclinic crystal structure of the Dps–DNA complex obtained in vitro [9]. It’s worth noting that our measurements were performed at pH 8.0 in the buffer containing 0.5 mM EDTA (ethylenediaminetetraacetic acid). The presence of EDTA in the solution is due to its potential to form a stable complex with divalent ions, e.g., Fe^2+^. Since Dps has some ferroxidase activity, the presence of a chelating agent is necessary to prevent DNA damage during the Dps–DNA complex formation [34]. In the work [9], for the first time, the multilayered Dps–DNA co-crystals exhibiting triclinic structure, which consisted of pseudo-hexagonal Dps layers alternating with DNA strands were visualized by CET. That served to determine the lattice parameters of the crystalline Dps–DNA complex and compared with SAXS data. Both data sets correlated well and were mutually reinforced, which, therefore, allowed us to build a detailed 3D model of the complex. However, our further investigations demonstrated that the Dps–DNA crystal structure depends on the parameters of the buffer in which the co-crystals are formed. Thus, in the present paper, we discuss the polymorphous behavior of Dps–DNA co-crystals and, for the first time, demonstrate Dps–DNA cubic structure obtained in vitro.

## 2. Materials and Methods

### 2.1. Preparation of DNA Sample

**Figure A1:**
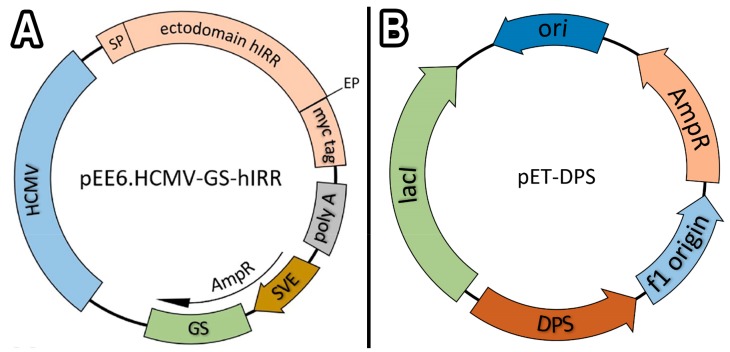
Map of pcDNA-IRR-GFP mammalian expression vector (**A**) and map of pET-DPS expression vector (**B**). (A) PCMV = promoter of human cytomegalovirus, SP = signal peptide, hIRR = cDNA encoding human IRR, EP = enteropeptidase cleavage site, GFP tag = tag at the C end, BGH pA = bovine growth hormone polyadenylation signal, SV40 = enhancer, Neomycin = a gene for resistance to neomycin, ColE1 = origin of replication, AmpR = ampicillin resistance gene. (B) DPS = cDNA encoding E. coli Dps, f1 origin = f1 bacteriophage origin of replication, AmpR = ampicillin resistance gene, ori = high copy number origin of replication, lacI = lactose operon.

Circular vector pcDNA-hIRR-GFP 9900 bp was used as the DNA sample (Figure A1A) [35]. Vector isolation on the silicon dioxide S5631 (Sigma-Aldrich Russia LLC, Moscow, Russia) was performed according to the protocol described in [36]. After isolation, this vector was precipitated with isopropanol again, washed with 70% ethanol, air-dried, and dissolved in Milli-Q water. Concentration of the DNA was determined by using the spectrophotometer ND-1000 (NanoDrop Technologies Inc., Wilmington, DE, USA).

### 2.2. Overexpression and Purification of Dps

Overexpression and purification of Dps was carried out according to a previously developed procedure [9]. The DNA fragment encoding Dps gene MSTAKLVKSKATNLLYTRNDVSDSEKKATVELLNRQVIQFIDLSLITKQAHWNMRGANFIAVHEMLDGFRTALIDHLDTMAERAVQLGGVALGTTQVINSKTPLKSYPLDIHNVQDHLKELADRYAIVANDVRKAIGEAKDDDTADILTAASRDLDKFLWFIESNIE (UniProtKB – P0ABT2 (DPS_ECOLI)) was obtained by PCR amplification of E. coli K12 MG1655 DNA, using forward 5′-GATATGAACATATGAGTACCGCTAAATTAG-3′ and reverse 5′-TATAAGCTTATTCGATGTTAGACTCGATAAAC-3′ oligonucleotides. The E. coli Dps gene was cloned into the expression vector pET-22b (+) (Figure A1B) at the NdeI and HindIII restriction sites. The nucleotide sequence of the recombinant gene, which was not modified by any tag, was checked by direct sequencing. Gene expression was carried out in E. coli BL21-Gold (DE3) cells grown in LB Medium, in the presence of ampicillin (150 μg/mL) at 37 °C. Transcription of the recombinant gene was induced by 0.5 mM IPTG at OD600 0.8, and accumulation of the protein was allowed for 4 h.

The protein was then purified by using ion-exchange chromatography on DEAE Sepharose FF column (GE Healthcare, Chicago, IL, USA) equilibrated with 20 mM of TrisHCl and 100 mM of NaCl, pH 7.5. The flow through fractions of proteins that do not bind to the sorbent were collected. These fractions contain the majority of the Dps protein, free from bound DNA [37]. The collected fractions were tested for DNA contamination by measuring the OD260/OD280, and this ratio is usually about 0.7. The protein was concentrated on Amicon ultrafiltration unit with a 10 kDa molecular weight cut-off and dialyzed again in the storage buffer containing 10 mM of Tris-HCl pH 7.5, 100 mM of NaCl, and 0.5 mM of EDTA. Protein purity was confirmed by SDS-PAGE. The purified Dps was aliquoted and stored at −20 °C.

### 2.3. Sample Preparation for Cryo-EM

The solution containing 1 mg/mL (4460 nM) of Dps and 3.1 mg/mL (482.48 nM) of DNA (9900 bp) was used for Dps–DNA co-crystals formation. 13.5 μL (13.5 μg, 60.21 pmol) of Dps protein and 4.5 μL (13.95 μg, 2.17 pmol) of DNA (9900 bp) in the concentrations corresponding to the Dps–DNA ratio 1 Dps dodecamer/345 bp of DNA were mixed with 2 μL gold nanoparticles solution (10 nm Colloidal Gold Labeled Protein A, UMC Utrecht, Netherlands) prior to CET study. Then, 3 μL of the mixture were applied to lacey carbon EM grid treated with a glow discharge (30 s, 25 mA) in Pelco EasiGlow. After blotting for 1.5 s at 10 °C, the grid with the specimen was plunge-frozen into a liquid ethane chilled with liquid nitrogen in Vitrobot Mark IV (FEI, Hillsboro, OR, USA). This procedure results in embedding the macromolecules (co-crystals) into a thin layer of amorphous ice, to preserve them in native state and to protect from radiation damages.

In our previous study [9] the influence of both Dps/DNA ratios and of DNA length on the process of the Dps–DNA co-crystal formation was studied in detail. Complexes with the following ratios were considered: 1Dps dodecamer to 345 bp of DNA; 1Dps dodecamer to 167 bp of DNA; 1Dps dodecamer to 66 bp of DNA; 1Dps dodecamer to 11 bp of DNA; 1Dps dodecamer to 5 bp of DNA. The most distinct peaks in SAXS curves belong to the composition with 1 Dps dodecamer/66 bp. Thus, this composition was used for further detailed SAXS study of the co-crystal structure and its polymorphism.

However, when we studied the solution with 1Dps dodecamer/66 bp of DNA in Cryo-EM, we found additional bulk nontransparent aggregates, which interfere with the co-crystal reconstruction. Several different compositions of DNA-Dps complexes were studied by Cryo-EM and we found the simultaneous formation of triclinic and cubic co-crystals but without aggregates in the 1 Dps/345 bp complex. Thus, we choose the complex composition as 1 Dps/345 bp much more suitable for cryo-EM. A constant buffer composition and Dps–DNA ratios of 1Dps/66bp and 1Dps/345bp resulted in identical types of co-crystals observed by Cryo-EM.

### 2.4. Cryo-Electron Tomography

The study was carried out with Titan Krios 60–300 TEM/STEM (FEI) CryoEM, equipped with TEM direct electron detector Falcon II (FEI), Cs image corrector (CEOS, Heidelberg, Germany) and VPP [38], at accelerating voltage of 300 kV. The accumulated total dose for VPP Cryo-TEM was ~30e/Å^2^, the defocus value was ~0.5 µm, and the VPP phase shift was ~π/2. For CET study, 15 datasets of the sample were collected automatically with FEI Tomography software in low-dose mode, with 18000x magnification, and the defocus value was in the range between –3 and –5 μm, using bidirectional tilt scheme (0°, –2°, …, -58°, –60°, 2°, 4°, …, 58°, 60°). The accumulated total dose was of 61e/Å^2^.

### 2.5. Tomographic Reconstruction

Cross-correlation alignment and tomography restoration were performed, using IMOD software, [39] by simultaneous iterative reconstruction technique (SIRT) and weighted back-projection (WBP) method. Gold nanoparticles were used as fiducial markers for the alignment of tilt-series projection images.

To find coordinates of centers of Dps molecules, tomogram segmentation was performed in an automated way, using convolutional neural network utility [40] in an open-source EMAN2.22 package [41] on 2 times binned data (pixel size 7.4 Å) restored with SIRT. Neural network was trained on Dps centers, manually picked from tomographic sections. Segmented tomograms were visualized in UCSF Chimera [42], and then clearly distinguishable co-crystals with plate-like morphology, described in [9], were manually erased from the dataset, which allowed us to focus on the possible new types of co-crystals. The illustration can be found in Video S1, where Dps molecules are highlighted by cyan color in cubic co-crystals and by pink in triclinic co-crystals.

To find coordinates of the intensity peaks on the segmented tomograms, corresponding to the Dps position, the reference-based boxing feature of EMAN2.22 was applied. A single Dps molecule in the center and several adjacent Dps molecules were chosen as a unit for the sub-tomogram averaging. The size of the sub-tomogram was 90px*90px*90px. Then, 55000 automatically picked sub-tomograms extracted from 15 unbinned WBP tomograms (pixel size 3.7 Å) were utilized for sub-tomo averaging in Relion2 [43,44], using the protocol described in Bharat et al. [45]. The 3D CTF model was obtained for each of the sub-tomograms based on the defocus value estimated with CTFFIND4 [46]. Two-dimensional projections of sub-tomograms, obtained with extraction feature “- - project3d” in Relion2, were used for 2D classification. After several rounds of 2D classification, coordinates of 42548 sub-tomograms were selected for further data processing.

To reduce the computational cost, the first step of 3D-auto-refinement was conducted on ~500 manually picked sub-tomograms. The obtained 3D map and 42548 sub-tomograms, remaining from the previous step, were utilized for reference-based 3D auto-refinement. It was observed that the contrast of the central Dps molecule in the sub-tomogram was higher than that of the others. Therefore, 3D classification was performed, and three classes of sub-tomograms were obtained: one with the uniform contrast of all particles; one with single Dps particle without any neighbors; one with central Dps particle on the edge of co-crystal (with adjacent Dps and DNA with the same density on one side and without any densities on the other side). Thus, it was concluded that the difference in contrast was caused by the presence of single Dps molecules and edge effects. Then, only one class, in which all the Dps molecules had the uniform contrast, was selected for further processing.

Afterward, 16,372 sub-tomograms were averaged by reference-free 3D auto-refinement. MTF-factor was taken into account for the post processing. Final resolution was estimated to be 13.5 Å using the 0.143 FSC criteria and 22 Å using 0.5 FSC criteria. Graphics, final visualization, and fitting were performed with UCSF Chimera [42]. The 1DPS structure from Protein Data Bank [15] and B-form helical DNA option in build structure feature in UCSF Chimera were used for fitting.

### 2.6. Solution Scattering Experiments and Data Analysis

Synchrotron SAXS measurements were performed at the European Molecular Biology Laboratory (EMBL) on the EMBL-P12 BioSAXS beam line at the PETRAIII storage ring (DESY, Hamburg) equipped with a robotic sample changer and a 2D photon counting pixel X-ray detector Pilatus 2M (DECTRIS, Switzerland, Baden). The scattering intensity, I(s), was recorded in the range of the momentum transfer 0.08 < s < 2.5 nm-1, where s = (4πsinθ)/λ, 2θ is the scattering angle, and λ = 0.124 nm is the X-ray wavelength [47]. The measurements were carried out in 10 mM of Tris-HCl, 100 mM of NaCl, and 0.5 mM of EDTA, pH 7.5, at 10 °C, using continuous sample flow operation over a total exposure time of 1 s, collected as 20 × 50 ms individual frames, to monitor for potential radiation damage (no radiation effects were detected) [48]. The data were corrected for the solvent scattering and processed using standard procedures with the program suite ATSAS [49]. Additional analysis of the repeating distances of the periodical motifs in the crystalline regions was performed as described elsewhere [9].

## 3. Results

### 3.1. Cryo-Electron Tomography

**Figure 1:**
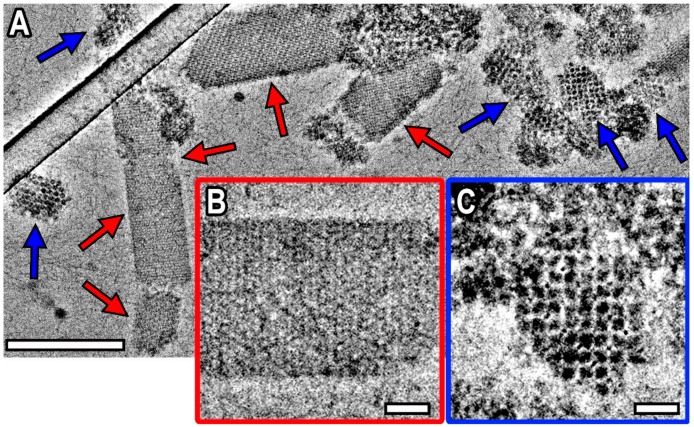
VPP Cryo-TEM images of Dps–DNA co-crystals. (**A**) An overview of the specimen, blue and red arrows indicate co-crystals with different crystal structure, bar is 200 nm; (**B**) an example of Dps–DNA co-crystal with triclinic crystal lattice, bar is 20 nm; (**C**) an example of the second type of Dps–DNA co-crystal, bar is 20 nm.

**Figure A2:**
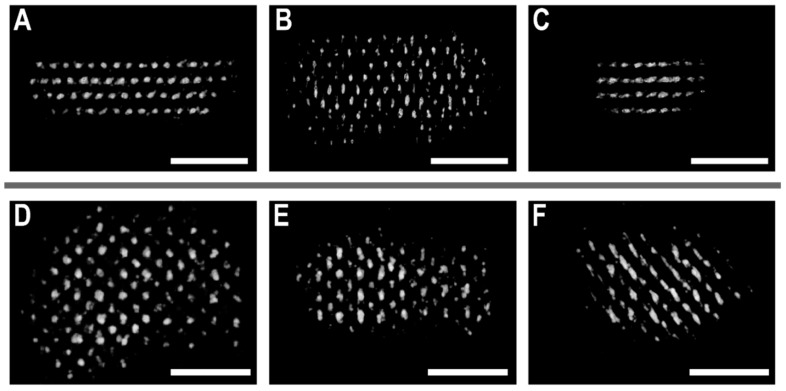
Triclinic (**A–C**) and cubic (**D–F**) Dps–DNA co-crystals in different orientations. White points indicate centers of Dps molecules on segmented tomogram. Bar is 50 nm.

**Figure A3:**
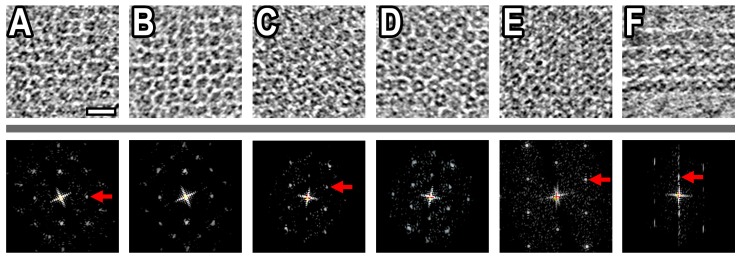
CET sections (top row) and corresponding FFTs (bottom row) of differently oriented cubic (**A**–**D**) and triclinic (**E**–**F**) Dps–DNA co-crystals: (A,C) 5 nm thick CET section of cubic co-crystal; (B,D) 20 nm thick CET section of cubic co-crystal; (E) 5 nm CET section of triclinic co-crystal; (F) 20 nm CET section of triclinic co-crystal. Bar is 20 nm. Red arrows on corresponding FFTs indicate peaks at 9.2 ± 0.3 nm (A,C), 7.9 ± 0.3 nm (E), and 10 ± 0.3 nm (F).

Figure 1A shows an example of Dps–DNA co-crystals formation visualized by Volta phase plate (VPP) Cryo-TEM. Two types of co-crystals (marked with red and blue arrows) with the size in the range between 40 and 300 nm were observed. Red arrows on Figure 1A indicate multilayered co-crystals exhibiting lamellar morphology (see Figure A2A–C and Figure A3E–F) with aspect ratio up to 10. Blue arrows indicate the second type of co-crystals, for the first time revealed in this study, which tend to be smaller and have aspect ratio not exceeding 2 (see Figure A2D–F and Figure A3A–D). Figure 1B,C shows the close-up view of the co-crystals. Besides, detached single DNA molecules are visible on Figure 1A, while free Dps molecules were not observed.

The detailed analysis and sub-tomogram averaging, performed on both types of co-crystals, confirmed that they possess different crystal structure. It’s worth noting that datasets for sub-tomogram averaging were collected from the single grid. However, the results proved to be reproducible since the Dps–DNA complexes with the same morphology and crystal structure were observed in several experiments conducted on different grids and with different vitrification parameters. The first type of co-crystals (red arrows in Figure 1A,B) exhibited triclinic crystal lattice (S.G. P1, with unit cell parameters determined by CET and SAXS a ≈ b = 9.3 ± 0.4 nm, c = 10.3 ± 0.4 nm, α = 73°, β = 90°, γ = 60°, EMD-4615) previously reported in [9].

**Figure 2:**
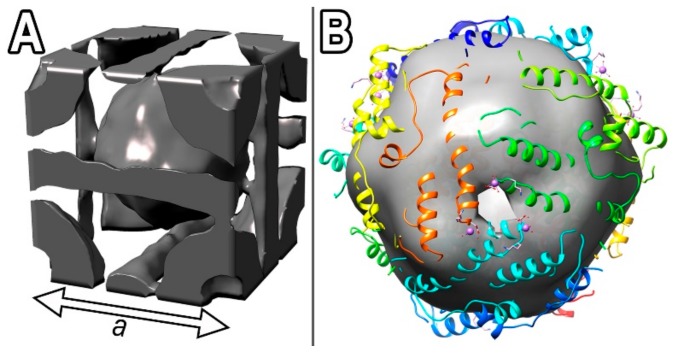
(**A**) Unit cell of the obtained cubic crystal lattice; (**B**) the 1DPS crystal structure [15] fitted into obtained CryoEM map. The smaller isosurface threshold of CryoEM map was chosen to reveal pores in Dps molecule.

**Figure 3:**
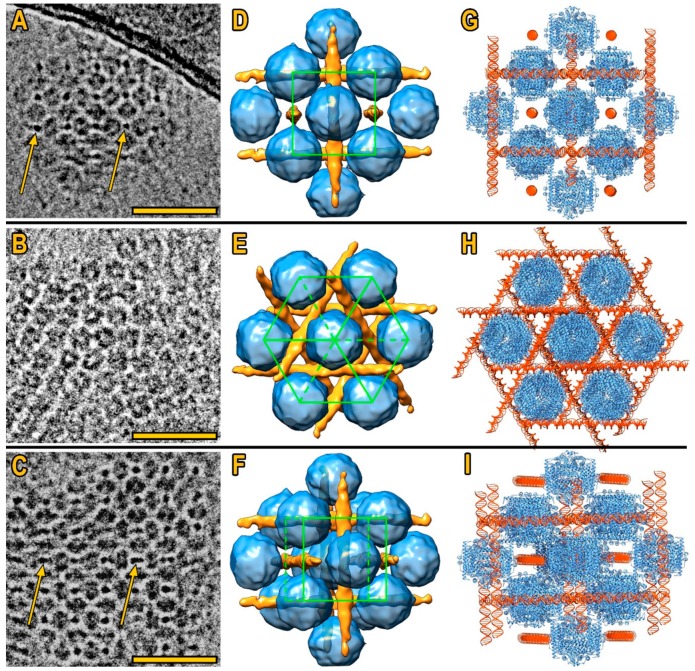
Cubic Dps–DNA co-crystals. (**A–C**) Cryo-TEM images of differently oriented Dps–DNA co-crystals ((A) **[001]**, (B) **[111]**, (C) tilted **[001]**), bar is 40 nm. Orange arrows indicate DNA molecules on Cryo-TEM images; (**D–F**) 3D reconstructions of co-crystals in corresponding orientations, Dps is shown in blue, DNA is shown in orange, the unit cell is highlighted by green; (**G–I**) known Dps and DNA structures fitted to the corresponding CryoEM densities.

**Figure A4:**
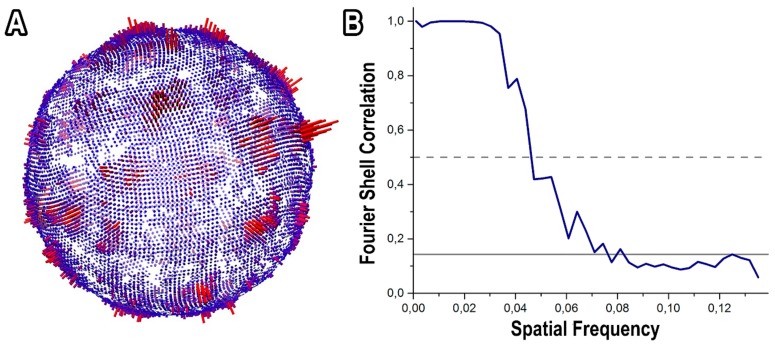
(**A**) orientation distribution of the Dps–DNA cubic co-crystals; (**B**) Fourier shell correlation (FSC) between two half-reconstructions of the Dps–DNA CryoEM map. Gray line—FCS = 0.143, and gray dotted line—FSC = 0.5.

The sub-tomogram averaging of the second type of co-crystals (see Figure 2A and Figure 3D–F) revealed that they adopted cubic crystal lattice (Figure 2A) and consisted of Dps molecules (Figure 2B), alternating with mutually perpendicular DNA strands (Figure 3). The space group of Dps molecules in the co-crystals appears to be Im$\bar{3}$m, but the DNA strands break the symmetry down to Pm$\bar{3}$m. The unit cell parameters were determined from Cryo-TEM images and CET sections of Dps–DNA co-crystals: a ≈ b ≈ c = 13 ± 1 nm, α ≈ β ≈ γ ≈ 90° and maximum interplanar distance 9 ± 1 nm. The error value was estimated by using full width at half maximum criteria of fast Fourier transform (FFT) spectra, obtained from the co-crystal images. It was established that, for each Dps molecule, there are 56 ± 3 bp of DNA in unit cell. The resolution was estimated to be 13.5 Å, using the 0.143 Fourier shell correlation (FSC) criteria (gray line, Figure A4B), and 22 Å, using 0.5 FSC criteria (gray dashed line, Figure A4B). Obtained electron density map was deposited to EMDB under the code EMD-10286.

The resolution of the obtained CryoEM map allowed us to visualize four acidic pores in the Dps molecule. One of these pores is visible in Figure 2B, and three others are located on the bottom part of the Dps molecule and cannot be seen in that projection. The pore locations lead to understanding of Dps orientation in the unit cell (Figure 2A). After the determination of unit cell parameters, it was established that it contains two Dps and three DNA molecules (Figure 2A). Each Dps molecule interacts with six DNA strands (Figure 2A). The observed acidic pores of central Dps molecule in unit cell are oriented towards the acidic pores of four Dps molecules in unit cell apexes, which are rotated by 90° relative to the **[001]** orientation of the central molecule.

**Figure A5:**
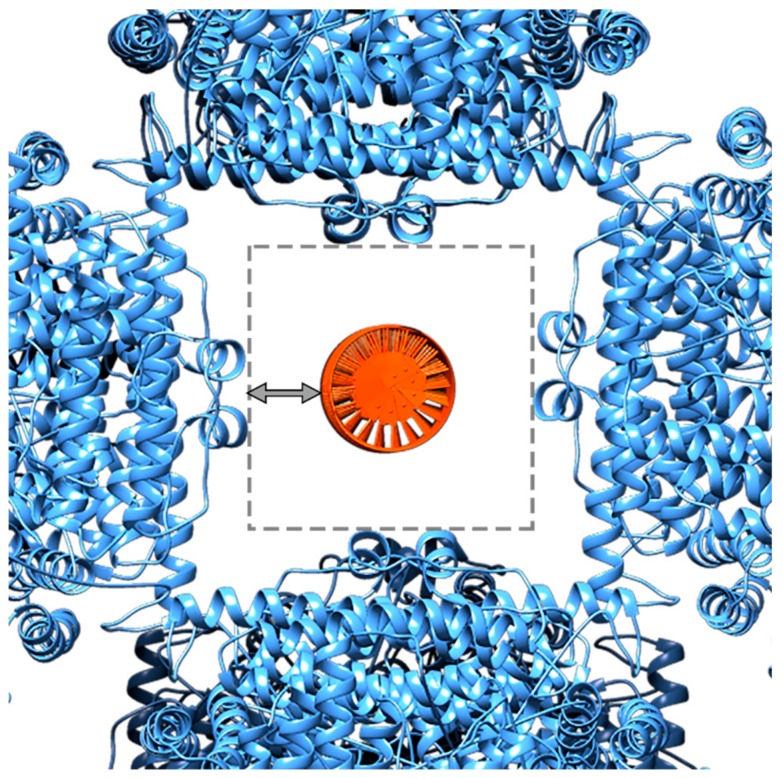
Illustration of free space between Dps and DNA molecules in cubic Dps–DNA co-crystals.

Figure 3A–C demonstrates Cryo-TEM images of co-crystals in different orientations (1st row **[001]**, 2nd row **[111]**, 3rd—tilted **[001]**). The 3D reconstruction of the corresponding orientations of co-crystals (Figure 3D–F) and models of 1 DPS [15] molecule and DNA fitted to the corresponding CryoEM densities (Figure 3G–I), where gaps of about 10 Å between Dps and DNA molecules can be observed (see Figure 3G and Figure A5).

The isosurface threshold for visualization (Figure 3D–F) was chosen based on the size of single Dps molecule taken from [15].

The fitted structure (Figure 3G–I) shows that E helix bundles [15] in each Dps are faced in direction of the DNA molecules. Each Dps dodecamer contains six pairs of E helix bundles located at the same distance from DNA (Figure A5).

### 3.2. Small-Angle X-Ray Scattering

**Figure 4:**
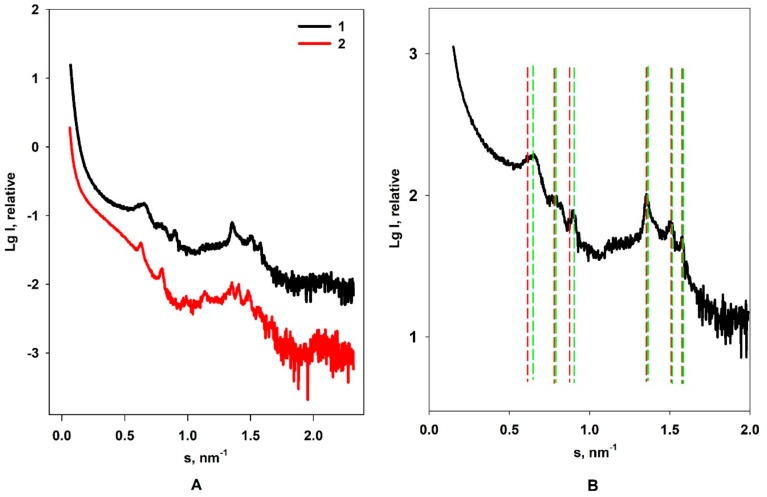
Experimental small-angle scattering curve of Dps–DNA co-crystals in solution at different buffers: 1 = pH 7.5, 10 mM of Tris-HCl, 100 mM of NaCl, and 0.5 mM of EDTA; 2 = pH 8.0, 50 mM of Tris-HCl, 50 mM of NaCl, and 0.5 mM of EDTA (**A**). The peak positions marked with lines depending on the type of crystal lattice at buffer 1 used in the present study (**B**). Red lines correspond to triclinic crystal lattice system (first peak corresponds to hkl = 001), and green lines correspond to cubic crystal lattice system (first peak corresponds to hkl = 110).

Additional characterization of the Dps–DNA complex structure was performed by small-angle X-ray scattering. To compare with our previous results [9], DNA with the length of 9900 bp was used. Figure 4A demonstrates the SAXS curves with characteristic peaks from Dps–DNA co-crystals in the solution at two different buffer compositions. The corresponding analysis of the positions s of the Bragg maxima on the SAXS curves and periodicity of the ordered motif d=2π/s was performed.

For the cubic phases, Bragg reflections occur at positions $s_{n}=2\pi \sqrt{n}/d_{1}$, $n=h^{2}+k^{2}+l^{2}$, where h, k, and l are integers. The cubic cell periodicity is either $d_{c}=d_{1}=2\pi /s_{1}$ (for the first observable reflection with Miller indices hkl = 100) or $d_{c}=d_{1}\sqrt{2}$ (for the first reflection with hkl = 110).

The optimal matching of the peak positions is obtained for spacing d = 13.9 nm, and the corresponding indices are given in Table 1.

As one can see from Table 1 and Figure 4B, the difference in maxima coordinates on the small-angle scattering curve for the cubic and triclinic lattice is negligible except for the first three Bragg peaks. Other peaks almost coincide. These results indicate that slightly different structures of co-crystals may coexist in the solution. Thus, the lattice parameters of the co-crystals obtained for the specimen with pH 8.0 buffer by SAXS perfectly fit the CET data.

Important characteristics of co-crystalline systems formed in the solution are integral parameters of small-angle scattering such as intensity at zero angle (the forward scattering *I(0)*) and excluded Porod volume (*Vp*) [50,51]. Comparison of these invariants, *I(0)* and *Vp*, calculated from SAXS curves for Dps–DNA complexes measured in two different buffers (Figure 4) shows that the values of these characteristics for the sample with the triclinic crystal lattice in the buffer 2 (*I(0)* = 490 ± 40; *Vp* = 5·10^5^ ± 1.5·103 nm^3^) are significantly higher than those for the co-crystals with the presence of cubic lattice in buffer 1 (*I(0)* = 170 ± 20; *Vp* = 3·10^5^ ± 1.2·10^3^ nm^3^).

Since these integral characteristics are proportional to the sample electron density, it should be concluded that the triclinic lattice is packed more densely. Again, there is good match between SAXS results and CET data, which demonstrated that the average density of triclinic Dps–DNA co-crystals is ~0.32 kDa/nm^3^ and that exceeds the cubic Dps–DNA co-crystals with the density ~0.23 kDa/nm^3^.

The size of the crystallites is also of great importance for the characterization of the Dps–DNA complex. The mean long order dimension, *L*, determining crystallite size, was calculated by using the Scherrer equation: $L=\frac{\lambda }{\beta_{s}\cos\theta_{1}}$, where $\beta_{s}$ is the full width at a half-maximum intensity of a peak (in radians) observed at a mean scattering angle of $2\theta_{1}$ corresponding to the momentum transfer $s_{1}$. This parameter was found to be 300–400 nm for the sample with triclinic packing, while for the cubic lattice the size of the crystallites was 100–150 nm. However, it should be emphasized that, when we consider triclinic or cubic packing in solutions of different buffer compositions, we mean crystal lattices, which are determined mainly by the first Bragg peak. At the same time, the peak widening, sample polydispersity and the coincidence of the positions of the secondary peaks on the scattering curves imply polymorphism of the Dps–DNA complex and coexistence of at least two different types of crystalline structures, with the predominance of one of the types in each of the solutions depending on buffer compositions. This section may be divided by subheadings. It should provide a concise and precise description of the experimental results, their interpretation, and the experimental conclusions that can be drawn.

## 4. Discussion

As we know, the protein–DNA complexes have not been reported to form mutually perpendicular DNA strands yet. However, structures which are similar to those reported in this paper have been found in the DNA-lipid complexes [52,53], and the morphology of the Dps–DNA triclinic structure found in the present and the previous [9] study is similar to one proposed by Ren et al. [33].

It could be that the polymorphous structures of Dps–DNA co-crystals appeared due to non-equilibrium conditions during solutions mixing. It should be remarked that the process of biocrystallization in vitro occurs in seconds, while in living cells it could take hours, days, and even months [54]. During rapid in vitro crystallization local heterogeneity of Dps and DNA molecules is springing up and mediating the difference in crystal structure.

It was previously demonstrated that Dps do not show canonic DNA-binding motif and negative charge prevails on the surfaces of both Dps and DNA molecules [15]. However, X-ray structure of Dps, which was demonstrated in that work, did not reveal unordered positively charged lysine-containing N-termini in 5, 8, and 10 positions.

While Ceci et al. [26] pointed out that symmetric spacing of N-termini on the dodecameric Dps surface leads to the formation of ordered crystals, similar to that in starved Escherichia coli cells, it was not assessed whether aggregation takes place through Dps–Dps or Dps–DNA interaction. We demonstrate that Dps molecules in cubic co-crystals interact with DNA rather than between themselves. Dps and DNA molecules are repulsed by electrostatic forces, which forms a ~1 nm gap between them (Figure 3G and Figure A5), while being connected by N-termini. Similar behavior was observed in our previous study [9]. Dps in the present CryoEM map is surrounded by six DNA molecules; therefore two N-termini could be involved in Dps interaction with single DNA molecule.

**Figure A6:**
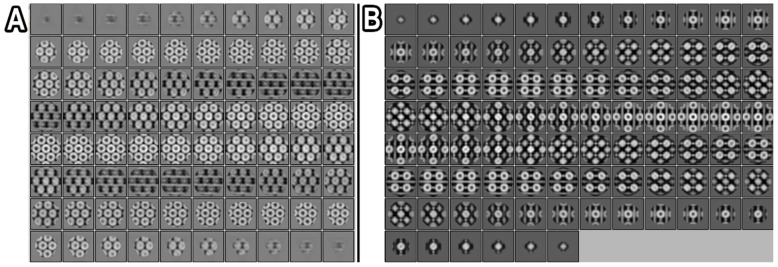
Two-dimensional slices of the CryoEM map of Dps–DNA co-crystals, adapting: (**A**) triclinic crystal structure, described in [15]; (**B**) cubic crystal structure, described in this article.

The difference in the amount of DNA per Dps dodecameric particle in cubic (56 ± 3 bp) and triclinic co-crystals (27 ± 3 bp) could be explained by structural differences: in cubic co-crystals, each Dps is surrounded by six DNA strands, while, in triclinic structure, each is surrounded by four DNA strands [9] (see Figure A6). This again indicates that a small difference in Dps–DNA ratio could lead to variations in crystal structure.

The 3D reconstruction of DNA molecules on Figure 3D–F demonstrates the linear region of DNA. Taking into account the average size of observed nanocrystals, the estimated length of such straight sector does not exceed 500 bp (out of 9900 bp DNA in total).

Previous in vivo studies suggested that Dps–DNA co-crystals consist of DNA strands alternating with pseudo-hexagonal packed Dps layers [1], which correlates well with the triclinic structure that was discussed in this and previous studies [9]. Besides, the values of the invariants and sizes of the crystallinity regions indicate that the triclinic lattice is more densely packed, while the cubic one is looser. From this, it could be assumed that the crystal lattices of the Dps–DNA complexes in living cells would be triclinic rather than cubic for more effective protection of cell genetic material. However, the polymorphous behavior of Dps–DNA co-crystals and the discovery of the cubic Dps–DNA crystal structure raises the question of the possibility of the formation of this type of co-crystals at different stages of biocrystallization.

## 5. Conclusions

This study demonstrated, for the first time, polymorphous behavior of Dps–DNA crystallization in vitro resulting in formation of cubic and triclinic structures depending on buffer parameters and local ion concentration. Co-crystals with central symmetric triclinic crystal lattice, which were described previously, have shown both Dps–Dps and Dps–DNA interactions, which lead to the formation of a multilayered microstructure. On the other hand, in the second type of co-crystals, for the first time, described in this paper, Dps–DNA interaction was observed, which lead to the formation of co-crystals with cubic crystal lattice (space group Pm $\bar{3}$m; unit cell parameters a ≈ b ≈ c = 13 ± 1 nm, α ≈ β ≈ γ ≈ 90°). Good correlation between CET and SAXS data completely supported the results of the co-crystal study.

## Figures and Tables

**Table 1:** Theoretical and experimental parameters of two types of the crystal lattice system.

Peak Position s, nm^–1^ (SAXS Data)	$\mathrm{Calculated}\;s_{hkl}\;$	$\mathrm{Calculated}\;d_{hkl}$	Miller Indices (hkl)
cubic crystal lattice system, a = 13.9 nm			
0.66 ± 0.02	0.64	9.81	$\left[110\right]$
0.80 ± 0.01	0.78	8.05	$\left[111\right]$
0.89 ± 0.04	0.90	6.98	$\left[200\right]$
1.35 ± 0.02	1.36	4.62	$\left[300\right]$,$\left[221\right]$
1.50 ± 0.01	1.50	4.19	$\left[311\right]$
1.57 ± 0.02	1.57	4.00	$\left[222\right]$
triclinic crystal lattice system, a = 9.1 nm; b = 9.5 nm; c = 10.4 nm; α = 75.2; β = 88.0; γ = 59.8			
0.63 ± 0.02	0.63	9.97	$\left[001\right]$
0.80 ± 0.01	0.80	7.85	$\left[100\right]$
0.89 ± 0.04	0.87	7.22	$\left[011\right]$
1.35 ± 0.02	1.35	4.65	$\left[300\right]$
1.50 ± 0.01	1.50	4.19	$\left[311\right]$
1.57 ± 0.02	1.57	4.00	$\left[222\right]$

## Supplementary Materials

The following are available online at https://www.mdpi.com/2218-273X/10/1/39/s1. Video S1: CET of the Dps–DNA co-crystals. The video demonstrates: slices through the tomographic volume; 3D segmentation results, where Dps molecules in cubic co-crystals are highlighted by cyan and in triclinic co-crystals by pink; slices through averaged sub-tomograms of triclinic and cubic co-crystals; 3D render of single Dps–DNA co-crystal with placed-back sub-tomogram averages (Dps molecules shown in cyan, DNA strands in red).
